# Editorial: Community series in the mechanism of trace elements on regulating immunity in prevention and control of human and animal diseases, volume II

**DOI:** 10.3389/fimmu.2023.1215080

**Published:** 2023-05-23

**Authors:** Dan Chen, Hao Wu, Xu Shi, Shiwen Xu, Ziwei Zhang

**Affiliations:** ^1^ College of Veterinary Medicine, Northeast Agricultural University, Harbin, China; ^2^ Key Laboratory of the Provincial Education Department of Heilongjiang for Common Animal Disease Prevention and Treatment, College of Veterinary Medicine, Northeast Agricultural University, Harbin, China; ^3^ Laboratory of Embryo Biotechnology, College of Life Science, Northeast Agricultural University, Harbin, China

**Keywords:** selenium, selenoproteins, macrophage, liver, lung, intestinal tract

Selenium (Se), an essential trace element for animals and humans, plays physiological functions mainly through selenoprotein, which deficiency would contribute to growth inhibition, reproductive disorder, and immune function dysfunction of body (1, 2). At present, 25 selenoproteins have been discovered in mammals and poultry, 24 of which exist as Sec-containing proteins, residing in virtually all tissues, exerting antioxidant, calcium homeostasis regulation and protein folding functions, as well as promoting the transduction and activation of various cellular signaling pathways (3). Notably, studies have found that selenium is involved in regulating immune function, and selenium deficiency would cause necrotizing lesions in immune organogenesis as well damage and abnormal differentiation of immune cells (4). A sociological survey results showed that the lack of diet selenium would give rise to body immune-incompetence which tended to increase susceptibility to infections, including inflammatory diseases and even developing into cancers in the gut and liver (5). Instead, researchers found that supra-nutritional levels of selenium intake elevated the levels of stress-related selenoproteins, which is in turn to affect the transcription and translation of genes involved in inflammation and interferon, improving the respiratory function of asthmatic mice (6, 7). Another successful example is that selenium level in the body regulates the proliferation and differentiation of T cells. The high Se diet skewed CD4^+^T to differentiate into Th1 phenotype, resulting in an immune-enhancing of mice and high IL-2 and IFN-γ levels in plasma (8). Additionally, there is some evidence that selenium also modulates the immuno-stimulatory, affecting T cell proliferation, NK cell activation and innate immune cell activity (9).

Macrophages are the components of innate immune system with cellular immunity, homeostasis maintenance and tissue repair, mainly derive from the monocytes in the bone marrow and exhibit extensive tissue distribution (10). Macrophages are capable to differentiate into two different phenotypes, namely pro-inflammatory M1 macrophages and anti-inflammatory M2 macrophages (11). A series of observations demonstrating that M1/M2 polarization balance is related to the pathogens-induced destructive reactions, as well repair after inflammation-associated injury, affecting anti-microbial defense, female animals pregnancy, anti-tumor immunity, metabolic disease and obesity, asthma and allergy, atherosclerosis, fibrosis, wound healing, and autoimmunity (12, 13). M1 macrophages are characterized by vigorous metabolism, engaging in the generation of high levels pro-inflammatory cytokines TNF-α, IL-1β, IL-6 and et al, and mediating the production of ROS involvement in pathogens removal, pro-inflammation and anti-tumor (14). The polarization shift of M1/M2 towards M1 direction would suppress LXA4/FPR, contribute to the activation of ROS/NF-κB/NLRP3 pathway, further governing the fate of pig lung fibrosis *via* TGF-β and PPARγ/Wnt pathways (15). Cytokines secreted by Th2 cells, such as IL-4 and IL-21, induce M2 macrophages polarization, driving M1/M2 to shift towards M2. Functionally, M2 macrophages have strong phagocytic ability, responsible for clearing debris and apoptotic cells, promoting tissue remodeling and coordinating wound healing (16). Studies found that M2 macrophages will be recruited to the wound area, whose released exosomes serve as a promoter of angiogenesis in vivo by regulating PTEN/AKT/mTOR pathway, conducive to tissue regeneration and shorten skin healing time (17).

As mentioned above, nearly macrophages in all tissues are affected by changes in the selenium status or selenoprotein expression (18). Although, for the most part, such common features including immunity, homeostasis and repair are shared by most macrophages, the functions of them also accompany tissue-specific due to differences in the internal environment of the organization (5, 19). The liver, with the largest population of tissue resident macrophages, is the main metabolic organ of the body (20). Thus, liver macrophages are more sensitive to dietary selenium (21). Researchers noticed that low-selenium diet imposed macrophages the identities of M1 and inhibited M2 phenotype activation, leading to liver fibrosis in rats (22). In contrary, selenium supplementation can inhibit liver necrosis by correcting M1/M2 imbalance, reducing the activity of M1 macrophages to diminish the release of inflammatory cytokines IL-1β and TNF-α, and decreasing the levels of key autophagy regulators ATG7 and LC3-II (23). CCL_4_ induced the accumulation of lipid peroxides and hepatocytes calcium overload in the liver, promoting the release of inflammatory cytokines in large quantities. Selenium supplementation could also keep the beneficial effects of macrophages to enhance antioxidant enzymes activity and reducing lipid peroxidation reactions, thereby inhibiting CCL_4_ induced liver injury in mice (24). Notably, the liver also has detoxification function and participates in the metabolism of selenium elements in the body, in which macrophages play a pivotal role. Thus for this, a lack of selenium-induced liver circulation disorders or immune stress are also associated with heart failure and downregulation of splenic cell proliferation (25, 26). In addition, selenium can indirectly cause thyroid atrophy, alopecia, and keratosis in goats by affecting the activation of macrophages in the liver (27). Mechanically, researcher found that the protective effects of selenium on liver are possibly attributed to the upregulation of antioxidant enzyme activity in macrophages and the inhibition of inflammatory factor secretion (28).

Pulmonary macrophages are derived from mononuclear cells in the blood, which are widely distributed in the interstitial lung, around the pipes below bronchioles and in the alveolar septum, and can migrate into the alveolar cavity. Pulmonary macrophages have very active functions of phagocytosis, immunity and production of various bioactive substances, which play an important defensive role (29). In addition, some studies have found that Se can change the interaction between macrophages and lymphocytes and play an antioxidant role for cells involved in immune response (30). Se-containing compounds can inhibit the release of inflammatory cytokines (TNF-α, IL-1β and IL-6, etc.) from macrophages caused by lipopolysaccharide-induced acute lung injury in mice (31). Se-dependent glutathione peroxidase plays an important role in the antioxidant defense of rat alveolar macrophages, such as the change of NADPH fluorescence of Se-deficient alveolar macrophages (32). Dietary Se deficiency can selectively inhibit leukotriene B4 (LTB4) biosynthesis in alveolar macrophages of lung rats. Compared with wild-type mice, mice lacking in macrophage selenoprotein M (TrspM) showed lower survival rate and increased bacterial load in lung and whole-body tissues, and the macrophages of lacking TrspM could not limit bacterial replication in vitro, which indicated that host selenoprotein might be considered as a new target for regulating immune response to control bacterial infection (33). Selenoprotein glutathione peroxidase-1 (GPX-1) knockout mice exposed to cigarette smoke increased the expression of IL-17A and MIP1α in the whole lung and the number of neutrophils and macrophages in alveolar lavage fluid, and regulated the expression of chemokine genes (34). To sum up, Se plays an important role in regulating the immune function and inflammatory response of pulmonary macrophages, and it may play a role through Se protein and antioxidant function.

The digestive system has the important task of absorbing nutrients and providing nutrients for the body, allowing nutrients to pass through and protecting the host from invading microorganisms. In order to maintain and regulate the stability of intestinal environment and function, there are one of the largest number of macrophages in the intestine. Low molecular weight selenomaminoglycan (LSA) significantly increased the gene expression levels of IL-2, IL-4, IL-10 and INF-γ in small intestine, and significantly increased the gene expression levels of IL-7β and iNOS in RAW264.7 cells. In short, LSA has immunomodulatory activity on immunosuppressed mice and macrophage RAW264.7 cells (35). After dextran sulfate (DSS) induced colitis in mice, supplementation of 0.4 ppm Na_2_SeO_3_ caused the inhibition of M1 markers (IL-1β, TNF-α) and the up-regulation of M2 markers (IL-10, Fizz1 and Arg-1) in colon tissue (36). Interestingly, conditional knockout mice with macrophage-specific Sec-tRNA^Sec^ (Trsp) showed that selenoproteins in macrophages were the key to avoid serious gastrointestinal injury (36). 0.8 ppm Se nanoparticles coated with ULP-SeNPs (Ulp-senps) has a significant protective effect on DSS-induced acute colitis in mice, improving macrophage infiltration, reducing CD68 and regulating the expression of cytokines IL-6 and TNF-α (37). To sum up, the deficiency of dietary Se or selenoprotein can aggravate the inflammatory and immune response in the intestine by affecting various signal pathways involved in oxidative stress and inflammatory response, which may be partly due to the fact that Se can polarize macrophages from the pro-inflammatory M1-like phenotype to the anti-inflammatory M2-like phenotype, thus reducing intestinal epithelial damage and inflammatory response.

Se is an essential trace element in the body, which covalently binds with amino acids in the body to form selenoprotein to show its biological function. The deficiency of Se or selenoprotein will cause immune dysfunction, morphological damage of immune organs and immune cell differentiation and dysfunction in humans and animals. Macrophages are regarded as the elves of human immune system, and they are antigen presenting cells that can play the functions of immune defense, immune homeostasis and immune monitoring. We found that Se and selenoprotein can regulate macrophages in liver, lung and intestine, including inflammatory reaction, M1/M2 polarization, antioxidation and cytokine expression. In the next research, we can start with Se supplementation or selenoprotein overexpression to explore the target and treatment strategy of macrophage-related immune diseases.

## Author contributions

DC: Writing–original draft. HW: Writing–review and editing. XS: Investigation, Visualization, References collection. SX: Writing–review & editing, Funding acquisition. ZZ: Supervision, Writing–review & editing. All authors contributed to the article and approved the submitted version.
